# Dental Items.—Are Vulcanite Plates Injurious?

**Published:** 1871-11

**Authors:** 


### EDITORIAL DEPARTMENT.

*Dental Items.—Are Vulcanite Plates Injurious?*—Dr H. W. Clarke writes on this subject to the "Am. Chemist," as follows:

"For two years past it has been my lot to lecture upon chemistry in the Boston Dental College. And, quite naturally, one of the questions which I am oftenest asked by unscientific friends is, whether the vulcanite plates upon which artificial teeth are set are injurious to health? The vulcanite being colored with vermillion, the popular prejudices against mercury are occasionally aroused with regard to it.

I have been in some measure forced to make inquiries in the matter. And for the benefit of those who may desire to know what little is known upon the subject, I have written the results of my inquiries.

In the first place, the question is not settled. Some hold that vulcanite plates are poisonous, while others believe them to be in all cases harmless. All I can do, then is to balance probabilities. Now, there are some cases in which the wearing of a vulcanite plate has been attended by sore mouth. But, on the other hand, similar soreness, some dentists assert, is frequently caused by gold plates. Unfortunately there are no statistics by which we can determine the relative values of gold and vulcanite as regards health. If it should be shown that gold plates produced soreness as often as vulcanite, then it would be clearly unsafe for us to ascribe the occasional ill effects of the latter to the mercury contained in it. It might be possible that some tender mouths would be irritated by any kind of plate.

Again, thousands upon thousands of vulcanite plates are worn, and the cases of injury are comparatively rare. Therefore it is well to bear in mind, in any case in which sore mouth might be ascribable to the mercury in the rubber, that it is quite possible that the soreness is due to some other cause. A man may have a sore mouth and wear a vulcanite plate, without the former being in any sense a consequence of the latter, There are such things as coincidences.

Taking all into account, then, it seems that there cannot be much danger in wearing vulcanite plates. That there *may* be some, I admit, but it is not proved that there is. It is certain that, in the vast majority of cases, there are no ill effect directly attributable to the mercury. Indeed, the metal is combined in such a form that it is hard to see how it should cause injury to any one. The sulphide is not likely to be attacked by the fluids of the mouth, and ought, theoretically, to be wholly inactive under the circumstances. Therefore, it seems to me, no person need reasonably be deterred from wearing a vulcanite plate by any fear of possible injury from it. The chances of injury, if any possibility of injury exists, are so slight that they may be practically disregarded. The most prudent people run greater risks of injury to health in their daily diet than they are ever likely to incur by wearing vulcanite.

